# LIS1 and DCX: Implications for Brain Development and Human Disease in Relation to Microtubules

**DOI:** 10.1155/2013/393975

**Published:** 2013-03-17

**Authors:** Orly Reiner

**Affiliations:** Department of Molecular Genetics, The Weizmann Institute of Science, 76100 Rehovot, Israel

## Abstract

Proper lamination of the cerebral cortex requires the orchestrated motility of neurons from their place of birth to their final destination. Improper neuronal migration may result in a wide range of diseases, including brain malformations, such as lissencephaly, mental retardation, schizophrenia, and autism. Ours and other studies have implicated that microtubules and microtubule-associated proteins play an important role in the regulation of neuronal polarization and neuronal migration. Here, we will review normal processes of brain development and neuronal migration, describe neuronal migration diseases, and will focus on the microtubule-associated functions of LIS1 and DCX, which participate in the regulation of neuronal migration and are involved in the human developmental brain disease, lissencephaly.

## 1. Introduction

Defined cell polarization is the key for the function of multiple cell types in the body, for example, the gut epithelium and the neuroepithelium, which both display an apicobasal orientation. Neurons, which part of them are generated from neuroepithelial cells, are also highly polarized cells with two distinct main structures that emerge from the cell body: usually a thin single axon, which is the key for signal transmission, and multiple shorter dendrites, which are designed for signal reception. The basic polarity of neurons was first recognized by Ramon y Cajal, who studied and described the morphology of neurons more than one hundred years ago [1]. In the cerebral cortex, two major types of neurons were defined: the excitatory or the glutaminergic neurons which compose the majority of the neurons in the cerebral cortex, and the inhibitory or the GABAergic interneurons composing the minority of the neurons. These two types of neurons are born in physically distinct areas of the brain, therefore, they need to migrate, sometimes very long distances, to reach their final destinations (reviews [2–6]). Genetic mutations, which affect polarity regulation and processes of neuronal migration in the developing brain, result in a wide array of human diseases. The range of diseases includes on the more severe end brain malformations, such as the lissencephaly-pachygyria spectrum, which defines the variety of diseases that cause relative smoothness of the brain surface and includes lissencephaly (smooth brain surface), agyria (no gyri), and pachygyria (broad gyri). In other cases, the brain surface may appear normal, but neurons can be mislocalized, which will be defined as cortical dysplasia. The position and the extent of the heterotropic neurons will further define the type of the brain malformation, for example; periventricular heterotopia (close to the ventricle), subependymal heterotopia (beneath the ependyma), subcortical heterotopia, or band heterotopia (neurons located in the white matter beneath the cortex as focal concentrations or a band) (reviews [7–13]). Patients with brain malformations will usually exhibit developmental delay, epilepsy, seizures, and intellectual disability depending on the severity of the brain malformation. These conditions usually can be diagnosed using brain imaging such as MRI (magnetic resonance imaging). However, neuronal migration diseases are not always detected by current noninvasive imaging techniques. Microscopic malpositioning of neurons could have been detected in many cases of childhood epilepsy following surgical procedures [14, 15]. Neuronal polarity and neuronal migration abnormalities are among the most common underlying primary defects in many cases of mental retardation or intellectual disability [16, 17]. Furthermore, autism and schizophrenia are also part of the spectrum of diseases involving neuronal polarity and neuronal migration regulation [18–21]. The prevalence of either mental retardation or schizophrenia is estimated to be about 1% of the population; the burden on society is huge since the patients live with the impairment over decades. Collectively, it can be appreciated that the wide range of neuronal polarity and neuronal migration diseases significantly impacts our society. Therefore, the understanding of the molecular mechanisms involved in these diseases, which also may affect therapeutic strategies, is of wide public interest.

Here, we will review the normal process of neuronal cell birth and migration, then will highlight the role of microtubules in this process, and will describe what happens when things go awry with special emphasis on the microtubule-associated functions of LIS1 and DCX.

## 2. Birth of Neurons of the Cerebral Cortex

**Figure 1:**
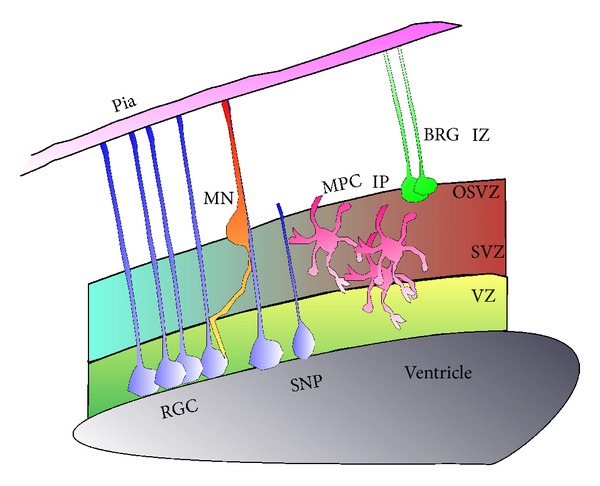
Schematic presentation of progenitors in the developing cerebral cortex. Radial glia cells (RGCs) extend their processes from the ventricular zone (VZ) to the pial surface. These cells proliferate in a symmetrical fashion to produce additional RGC or asymmetrically to produce a progenitor and a multipolar cell (MPC), which may be either a postmitotic neuron or an intermediate progenitor (IP), which can further divide in the subventricular zone. The VZ contains additional short neuronal progenitors (SNP). In the SVZ and the outer SVZ (OSVZ), an additional type of progenitors was described, basal radial glia (BRG), which lack the connection to the ventricle. A bipolar migrating neuron (MN) is moving towards the intermediate zone (IZ).

**Figure 2:**
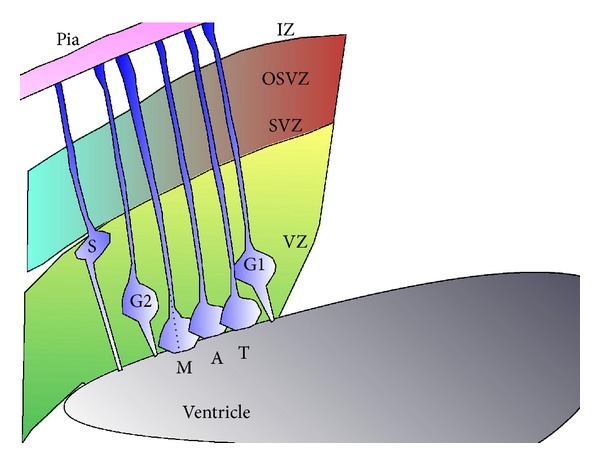
Schematic presentation of interkinetic nuclear movements in the ventricular zone (VZ). Nuclei of RGC are found at the upper surface of the VZ during S phase (S). The nuclei of the cells undergoing mitosis (M phase) are located close to the ventricle, where they complete anaphase (A) and telophase (T). Nuclei in G1 and G2 phases are found in intermediate positions.

The majority of neurons in the cerebral cortex, the pyramidal or the excitatory neurons, are born either within the ventricular zone or the subventricular zone (reviews [22–26]). During early development, neuroepithelial cells proliferate mainly to generate additional progenitors (reviewed [22, 26]). Later, two types of progenitors in the ventricular zone are defined; most of them are the radial glial cells that span the entire neocortical wall and maintain contact both at the ventricular and pial surfaces throughout mitotic division, and the minority of them are the short neural precursors that possess a ventricular endfoot and a basal process of variable length that is retracted during mitotic division [27, 28] (Figure 1). The radial glia is the major population of neural progenitor cells occupying the proliferative ventricular zone in the developing mammalian neocortex [29–31]. Radial glia cells serve as progenitors in all regions of the central nervous system [32]. Radial glia cells exhibit typical interkinetic nuclear migration, where the nucleus moves within the cytoplasm of elongated neuroepithelial progenitor cells in synchronization with the cell cycle phase [33, 34] (review [35]). The nucleus ascends to the upper region of the proliferative zone, the ventricular zone, during S phase and later descends to the apical part of the ventricular zone (Figure 2). Mitosis is restricted to the most apical regions of the ventricular zone after the nucleus completed its descent. The variable positioning of the nucleus within the ventricular zone is the basis for the pseudostratified appearance of the progenitor-cell layer known as the ventricular zone (Figure 2).

The radial glia precursor stem cells undergo symmetric and asymmetric divisions while producing large numbers of diverse cortical cell types. However, the relative orientation of the cleavage plane does not define whether the divisions are symmetric or asymmetric [36, 37], but rather the relative inheritance of the apical plasma membrane dictates whether the daughter cell fate will be symmetric or asymmetric [38–42]. Asymmetric divisions will result in self-renewal of the progenitors, but will also produce more committed daughter cells, intermediate progenitor cells (also known as basal progenitors), outer subventricular zone progenitors, or postmitotic neurons [43] (review [24]). During development, there is a gradual shift from proliferative divisions to neurogenic divisions. This has been accompanied with progressive lengthening of the cell cycle [44]. Further studies indicated that artificial lengthening of the cell cycle can be sufficient to switch neuroepithelial cells from proliferative to neurogenic divisions [45, 46].

The second proliferative area for excitatory neurons of the cerebral cortex is the subventricular zone. Within this area, progenitors usually divide in a symmetrical way, and in contrast with the radial glia, their processes do not make contact with the apical or pial surfaces. These intermediate or basal progenitors are daughter cells of either neuroepithelial or radial glial cells located in the ventricular zone [43, 47–49] (reviews [22, 24, 50]). Mitotic intermediate progenitor cells are largely found in the subventricular zone, but it should be noted that they can also undergo division in the ventricular zone and the intermediate zone [51, 52]. Typical intermediate progenitors in the developing neocortex usually undergo one terminal symmetric division that produces two neurons, while some cells undergo two rounds of symmetric divisions (Figure 1).

In primates and humans, the subventricular zone is widely expanded and they develop an additional proliferative region known as the outer subventricular zone [53–55]. Cell-labeling studies in primates have shown that cell divisions in both the outer subventricular zone and the ventricular zone coincide with the major wave of cortical neurogenesis, suggesting that outer subventricular zone cells produce neurons [56, 57]. The outer subventricular zone was found to contain two types of progenitors: radial glia-like cells and intermediate progenitor cells [58, 59]. The outer subventricular zone radial glia-like cells have a long basal process; however, the contact with the ventricular surface is lacking. These cells undergo proliferative divisions and self-renewing asymmetric divisions to generate neuronal progenitor cells that can proliferate further and can also generate neurons. Initially it was thought that these cells exist only in primates, but similar progenitors have also been observed in nonprimates, such as ferrets [59] and mice [40, 60].

**Figure 3:**
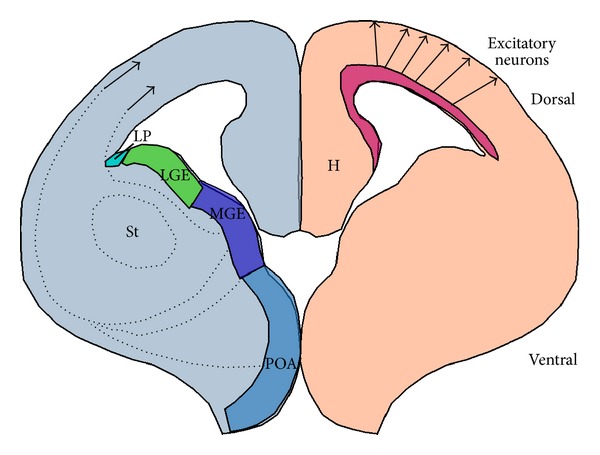
Schematic presentation of migratory routes of excitatory and inhibitory neurons in the developing brain (adapted from a review by Tan and Shi, *WIREs Dev Biol *2012. doi: 10.1002/wdev.88). Inhibitory neurons are shown in the left side. Most of the GABAergic neurons are born in the ventral part of the telencephalon, in the subpallium, and more specifically in the medial ganglionic eminence (MGE) and the lateral ganglionic eminence (LGE), in the subpallial septum, and the embryonic preoptic area (POA), and they migrate in a tangential way to the cortex. St: striatum; LP: lateral pallium; H: hippocampus.

The inhibitory neurons, or the GABAergic neurons, are born in a different position than that of the excitatory neurons. Most of the GABAergic neurons are born in the ventral part of the telencephalon, in the subpallium [61] (for reviews see [62–64]). More specifically, the medial ganglionic eminence (MGE) and the caudal aspect of the lateral ganglionic eminence (cLGE) (also known as the dorsal aspect of the caudal ganglionic eminence (dCGE)) generate most of the cortical GABAergic interneurons [65]. However, additional sources of cortical GABAergic interneurons are the rostral LGE, the subpallial septum, and the embryonic preoptic area (POA) [66–68] (Figure 3). Radial glia cells are progenitors for inhibitory neurons as well as for excitatory neurons [32] (for reviews see [69–71]). These progenitors not only share structural similarities, but the ventral progenitors in the MGE were shown to also undergo asymmetric cell divisions to produce neocortical interneurons [72]. Furthermore, neocortical inhibitory interneurons were produced as spatially organized clonal units in the developing ventral telencephalon. However, although the radial glia cells in different areas of the cerebral cortex appear morphologically similar, they do not share the same molecular identity. Radial glia cells in the ventricular zone of the telencephalon express the transcription factors Pax6 and Emx1 [73, 74]. In their absence, the cells convert their fate to that of ventral telencephalon cells [75]. Radial glial cell in the ventral telencephalon express different sets of transcription factor genes, for example, Gsx1/Gsx2 and Olig2 [73, 76, 77]. In addition to proliferating radial glial cells, the ventral telencephalon contains multiple intermediate progenitors, which divide symmetrically to produce interneurons [76, 78]. These proliferating progenitors are an important source of the interneurons since the subventricular zone in the ventral telencephalon is larger than that in the neocortex.

## 3. Migration of Neurons of the Cerebral Cortex

As mentioned above, neurons are usually born in a position, which differs from their terminal destination. Thus, neurons need to migrate from their place of birth to their final position using several types of cellular motility (reviewed [2, 4, 6, 79–82]). The position of neurons within defined layers of the cerebral cortex is dependent upon their birth date and their proper movement from their place of birth to their accurate placement. The six layers of the cerebral cortex are composed of neurons that are born in different areas but are subsequently organized according to their birth dates [83, 84]. Interestingly, this organization is not unique to excitatory neurons. Many interneurons and excitatory neurons that are born at a similar time end up occupying the same neocortical layer; however, little is known how this amazing coordination is achieved [85–88] (review [69]). Neurons born relatively late during corticogenesis reside in more superficial layers on top of the older neurons, thus composing an inside-out organization. Early in development, these cells usually move using cellular locomotion. Later, neurons migrating along this route attach to radial glia, which provide a transient scaffold for directed migration [2–4, 89, 90]. Neurons migrating along radial glia exhibit a bipolar structure. Once these cells reach the pial surface or their correct position, they detach from the radial glia and continue to move towards their correct laminar position. A different mode of migration, known as tangential migration, is employed by the interneurons, which migrate tangentially across the plane of the glial fiber system [2, 4, 80, 85, 91]. Once they reach the cerebral cortex, they employ the radial route and migrate along radial glia to their proper laminar position [66, 92–100] (routes of migration are schematically shown in Figure 3). Thus, even if only the radial route of migration is disrupted, the position of inhibitory neurons in the cerebral cortex will be affected, since they use both the tangential and the radial route.

Newly formed neurons in the ventricular zone undergo an initial morphological transition to a pin-like structure following their final mitoses [101]. These cells are still lacking a leading edge, and their centrosomes are localized towards the ventricular endfoot. The ventricular endfoot is then retracted, and the cells adopt a multipolar structure where cells extend neurites in multiple directions. Live imaging of *in utero* transfected cells reveals that each of the radially migrating neurons undergoes this complex morphological transition [4, 102, 103]. It has become apparent that this transient morphology is particularly sensitive to genetic manipulations, as knockdown of several genes resulted in accumulation of stalled neurons with multipolar morphology (reviews [104, 105]). The multipolar stage is transient and is followed by acquirement of bipolar morphology [43]. Neurons redefine their polarization; they first extend an axon, which will be the future trailing edge, orient the centrosome in front of the nucleus, and generate a leading edge, which has some characteristics of a dendrite [106]. In radially migrating cerebral neurons, the centrosome moves in a very processive manner, whereas the nucleus, which composes most of the cell body, follows this movement in a stepwise manner [107–110]. Failure to translocate the nucleus will translate to abnormal migration and will affect the proper laminar organization of the developing cortex. Thus, during the normal course of migration, the cells will change their morphology to a bipolar one and continue to migrate along radial glia in an ordered fashion (Figure 1). Therefore, acquisition of polarity is imperative both for initiation and continuity of directed motility of neurons to their targets.

## 4. Neuronal Microtubules and the Centrosome

The morphological changes that take place in migrating neurons require coordinated regulation of the cytoskeleton (review [18]). We will focus on the microtubules, which are key components of the neuronal cytoskeleton. Microtubules are long cellular polymers composed of subunits of alpha- and beta-tubulin. They exhibit dynamic instability, which can be visualized both *in vitro* [111, 112] and *in vivo* [113–115]. Microtubules exhibit an inherit polarity, where tubulin subunits are preferentially added to the plus ends. The microtubule cytoskeleton participates in structuring of the cell and provides directional rails for transport of intracellular organelles and different cargoes (review [116]). In neuronal progenitors and in early born neurons, most of the microtubules emanate from the microtubule organization center, the centrosome, and its proper function and intracellular localization are believed to be of great importance both in proliferating and migrating cells (reviews [18, 35, 81, 105, 117, 118]).

In polarizing neurons, it has been proposed that the position of the centrosome may predict the location of the future axon [119, 120]. However, this notion has been questioned following several findings. In neurons from the tegmental hindbrain nuclei in zebrafish, axon outgrowth occurs at clear distance from the centrosome [121]. Probably, an earlier cue for polarization is the relative position of N-cadherin. Endogenous N-cadherin was found to localize to one pole of the newborn neuron, from where the first neurite will emerge [122]. The Golgi and centrosome move towards this newly formed morphological pole in a second step, which is regulated by PI3 K (phosphoinositide 3-kinase) and the actin/microtubule cytoskeleton.

In mature neurons, the proportion of acentrosomal microtubules is significantly higher (review [117]). In contrast with migrating neurons, the axons of mature neurons contain microtubules that form a continuous array, extending from the cell body into the growth cone at its distal tip (comprehensive review [123]). It has been shown that the centrosome loses its function as a microtubule-organizing center in developing hippocampal neurons [124]. Most importantly, microtubule arrays in mature neurons are highly organized in regard to their intrinsic polarity. Early studies have determined the orientation of microtubules in mature neurons by electron microscopy-based techniques [125]. Most of the microtubules in the axon are oriented with their plus-ends facing the growth cone, while in the dendrites the microtubules are oriented in both directions. Thin and distal dendrites exhibit unipolar microtubule orientation similar to the axonal ones (review [116]). Live imaging using a fluorescently tagged plus-end tracking protein (EB3-GFP) confirmed these findings and enhanced the capability to rapidly evaluate the orientation of growing microtubules in culture and *in vivo* [126].

## 5. Neuronal Migration and Brain Developmental Deficits in Humans

Deficits in neuronal migration in humans have provided us with insights on the regulatory mechanisms involved in this process. Abnormal neuronal migration may result in cortical malformations, and in extreme cases, the brain is smooth (lissencephalic) lacking most of the normal typical brain convolutions. Lissencephaly (i.e., smooth brain) is a severe human neuronal migration disorder (review [127]). Based on brain histology; two types of lissencephaly were defined: type I or classical lissencephaly and type II or cobblestone lissencephaly [128]. Whereas in type I the cerebral cortex consists of four layers, in type II no discrete layers are formed. Moreover, in type II lissencephaly, the manifestation of the phenotype including the cerebellum and the brainstem as well as other organs, such as eyes and muscles, has been noted [129]. Disorganization of the cortical layers reflects mainly migration deficits in excitatory neurons. Yet it should be noted that the migration of inhibitory neurons is also diminished [130]. Imbalances between excitatory neurons and inhibitory neurons are one of the underlying causes of epilepsy. Therefore, it is not surprising that epilepsy is a common feature among lissencephalic patients [131].

Mutations in several genes have been associated with type I lissencephaly, among them *lissencephaly-1 *(*LIS1*) [132], the X-linked gene *doublecortin *(*DCX*) [133, 134], and *tubulin alpha 1A *(*TUBA1A*) [135–137]. The phenotype is characterized by absent (agyria) or decreased (pachygyria) convolutions, producing a smooth cerebral surface with thickened cortex [138]. Some differences were noted in cases of lissencephaly due to mutations in *LIS1 *versus mutations in *DCX. *In case of *LIS1 *mutations, the brain is more affected in the dorsal portion of the brain, whereas *DCX *mutations affect the more rostral part [139, 140]. Furthermore, a limited study of two fetal brains, one mutated in *LIS1 *and the other mutated in* DCX,* revealed differences in the histology [141]. In the *LIS1* mutated brain, the cortical ribbon (the grey matter) displayed a characteristic inverted organization, also called “four-layered cortex”, while in the *DCX* mutated brain, the cortex displayed a roughly ordered “six-layered” lamination. It was suggested that additional studies, especially of *DCX* mutant, brains may help to clarify this issue. Subcortical band heterotopia (SBH) is a related disorder in which there are bilateral bands of gray matter interposed in the white matter between the cortex and the lateral ventricles. SBH (double cortex) is very common among females with mutations in DCX [133, 134]. SBH can also be observed in cases of somatic or mild mutations in LIS1 [142, 143]. Lissencephaly and SBH have been observed in different regions of the same brain, defining an “agyria-pachygyria-band” spectrum [140]. Mutations in *TUBB2B *have been associated with a different brain malformation, asymmetrical bilateral polymicrogyria [144]. Polymicrogyria is characterized by a disorganized cortical lamination and the presence of multiple small, partially fused gyri separated by shallow sulci that produce an irregular cortical surface [145]. A partial duplication of LIS1 has been detected in a patient with microcephaly (reduced brain size), neurodevelopmental delays, and profound white matter atrophy in the absence of lissencephaly [146]. All of the protein products of the genes mentioned above, tubulin, LIS1, and DCX are involved in formation and regulation of microtubules, which play an important role in the developing brain.

Less noticeable deficits in neuronal migration are responsible for a significant proportion of cases of mental retardation and epilepsy in children [8, 14, 147]. Furthermore, it is proposed that abnormal neuronal migration also plays a role in schizophrenia and autism spectrum disorders (ASDs) (reviews [148–150]). Thus, there are multiple examples indicating a strong link between intellectual disability and abnormalities in neuronal migration [2, 105]. *MARK1/Par1* has been proposed as a susceptibility gene for autism [151]. MARK (microtubule-associated protein/microtubule affinity-regulating kinase) composes a small family of proteins [152], which were first identified to regulate the dynamics of microtubules [153, 154]. Increased dosage of LIS1 also impairs processes of normal brain development and results in delayed development, mental retardation, and autism [155–157]. One of the reported patients with increased LIS1 dosage exhibited epileptic seizures, which fitted the diagnosis of classical West syndrome [131].

In addition, microdeletions of a region encompassing the *MAPT* (microtubule-associated protein tau) gene, encoding for the tau protein, result in moderate mental retardation with associated dysmorphic features [158–162]. The frequency of the microdeletion syndrome was estimated to be 1 : 13,000 to 1 : 20,000, thus suggesting it to be a common underlying cause for mental retardation. *MAPT *is one of the few genes within the microdeletion locus; it is strongly expressed in the developing brain [163, 164], and it has been suggested to play a role in neuronal migration. Tau is a well-studied, brain enriched, microtubule-associated protein, which was initially identified by virtue of its capability to enhance microtubule polymerization *in vitro* [165, 166]. Tight regulation of the dynamic instability of microtubules allowing for rapid transition between the growing and shrinking phases is essential for proper neuronal migration. Furthermore, it should be noted that regulation of proper microtubule dynamics and axonal transport plays an important role in multiple neurodegenerative diseases; this topic has been extensively reviewed and is not the focus of this current review (e.g., [123, 167–174]).

## 6. LIS1 and DCX: Microtubules and Cellular Polarity

A tight relationship between LIS1, microtubule regulation, and microtubule-based motor proteins has been demonstrated in many organisms. The first functional insights on LIS1 and their role in regulation of microtubules and microtubule-based motors came from studies conducted in a fungus, *Aspergillus nidulans*, during a screen conducted by the Morris lab. Fungi mutated in LIS1, designated in *Aspergillus nidulans* as nudF (nuclear distribution gene F) exhibited impairments in the ability to move nuclei along microtubules in the growing hyphae. Interestingly, a mutation in the alpha-tubulin gene suppressed mutations in nudA (dynein heavy chain), nudC (LIS1 interacting protein), nudG (dynein light intermediate chain) and nudF [175]. We have shown that LIS1 interacts with tubulin and modulates microtubule dynamics *in vitro* [176], suggesting an evolutionary conserved LIS1 function. The role of LIS1 in preserving the normal microtubule network organization *in vivo* has been shown both in mammalian cells [177–179] and in cells of the simple organism *Dictyostelium* [180]. LIS1-microtubule interaction and probably other LIS1 interactions are regulated by phosphorylation [181]. In addition to a direct role for LIS1 in regulating tubulin dynamics, LIS1 interacts with a plethora of microtubule-associated proteins (MAPs). This includes interactions with DCX [182], CLIP-170 [183], and MAP1b [184]. Furthermore, LIS1 may affect actin polymerization through Cdc42 and IQGAP [185, 186].

An evolutionary conserved function of LIS1 in regulation of cytoplasmic dynein was first noted in the fungus, *Aspergillus nidulans*. Three of the *Aspergillus nidulans* nud genes (nudA, nudI, and nudG) are subunits of cytoplasmic dynein, a microtubule-based motor protein, and a fourth gene, nudK, is a part of the dynein regulatory complex dynactin [187–189]. Genetic interaction of LIS1 with dynein/dynactin/microtubule-mediated pathway has also been suggested from work on early development in *Drosophila* [190–192], demonstrating that LIS1, like dynein heavy chain, is essential for germ-line division, nuclear positioning, and oocyte differentiation. Moreover, also in *Saccharomyces cerevisiae*, the LIS1 ortholog was found to be involved in nuclear migration [193], which is mediated by microtubules and regulated by the dynein pathway [194]. The evolutionary conservation of LIS1 with the dynein pathway has been extended to mammals, where it interacts with several subunits of the retrograde, microtubule-based motor complex dynein/dynactin [177, 178, 195, 196]. It regulates cytoplasmic dynein activity [177] and participates in several dynein-mediated activities, such as intracellular transport [197, 198], organization of intracellular organelles [199–201], and mitosis [195, 202–204].

In contrast to other known proteins interacting with dynein, LIS1 binds to dynein motor domain [205] (review [206]). LIS1 was found to strengthen the dynein-microtubule interaction [205, 207] (reviewed in [208]). In addition, it has been proposed that LIS1 may have a role in initiating dynein-driven motility [209]. Single-molecule laser bead trap analysis revealed that LIS1 substantially prolonged dynein stalls under load [207]. With LIS1 bound, dynein is arrested in a strongly microtubule-bound state, although ATP hydrolysis can still go on [205].

LIS1 and cytoplasmic dynein play an important role in the regulation of the polarity of microtubules. As mentioned above, axonal microtubules are normally oriented uniformly plus-end-distal; however, without dynein or LIS1, axons contained both plus- and minus-end-distal microtubules [210]. Probably this mixed polarity of the microtubules allowed for the entry of dendritic organelles and proteins to the axon [210].

LIS1 itself has been shown to be a cargo for the anterograde motor Kinesin-1[211, 212]. Kinesin-1 interacted with the NUDEL (nuclear distribution protein nudE-like 1)/LIS1/14-3-3epsilon complex through DISC1 (disrupted in schizophrenia 1), and interference with the complex affected protein localization and axonal outgrowth.

DCX was characterized as a microtubule-associated protein (MAP) [213–215] shortly following its discovery as a gene mutated in cases of X-linked lissencephaly [133, 134]. DCX promotes nucleation and the assembly, and stability of 13-protofilament microtubules [216, 217]. DCX interacts with the sides of the lattice of microtubules and stabilizes the 13-protofilament structure through a cooperative interaction, wherein DCX molecules decrease the dissociation rate of their neighbors [218]. DCX is part of a small superfamily of proteins, characterized by the existence of one or two conserved DCX domains [219, 220]. Interestingly, most mutations that are found in lissencephaly patients cluster in the well-defined DCX domains [221, 222]. We have shown a physical interaction between LIS1 and DCX, and *in vitro* both proteins enhance tubulin polymerization in an additive manner [182]. A genetic interaction between LIS1, DCX, and cytoplasmic dynein has also been suggested [223]. DCX is also involved in the regulation of the actin cytoskeleton, in a direct and indirect way [224–226]. Additional interactions have been observed between DCX and mu subunits of clathrin adaptor complexes [227]. In developing cultured neurons, DCX modulated endocytosis and thereby the surface distribution of neurofascin. Interestingly, this activity has been shown to be independent of DCX's interaction with the microtubules [228].

The interaction between DCX and microtubules is regulated, at least in part, by phosphorylation. DCX is phosphorylated by at least four different kinases: JNK [229], Cdk5 [230, 231], protein kinase A (PKA) and/or the MARK/PAR-1 family of protein kinases [232], and GSK3*β* [233]. Protein phosphatase 1 (PP1) has been shown to dephosphorylate some of the sites [234, 235]. *In vitro *analysis indicated that DCX's phosphorylation by Cdk5, PKA, and MARK reduced the affinity of DCX to microtubules [230, 232]. Phosphorylation of DCX by different kinases yielded different outputs; Cdk5-mediated phosphorylation inhibited the ability of DCX to promote microtubule bundling and suppressed axon branching [230, 234], while GSK3*β*-induced phosphorylation of DCX promoted the function of DCX in the regulation of axon branching and self-contact [233]. The effect of DCX on branching may be associated also with its interaction with the actin cytoskeleton. DCX depletion significantly delayed collateral branching in hippocampal neurons and also significantly lowered the frequency of actin-rich patches along hippocampal axons [236]. DCX not only affects the development of the axon but also the dendritic arborization [237].

Microtubules stabilized by DCX are preferred substrates for kinesins [238]. Furthermore, DCX has been recently found to associate with a member of the kinesin superfamily, namely Kif1a, which is a Kinesin-3 molecular motor protein that traffics synaptic vesicles [239]. Neurons lacking *Dcx* and/or its closed family member, *doublecortin-like kinase 1 (Dclk1)*, showed impaired Kif1a-mediated transport of Vamp2, a cargo of Kif1a [239]. The same study demonstrated that DCX specifically enhanced binding of the ADP-bound Kif1a motor domain to microtubules.

## 7. The Roles of LIS1 and DCX in Processes of Brain Development

### 7.1. Neurogenesis in the Developing Brain

LIS1 levels affect cell proliferation in the developing brain at multiple stages [108, 155, 240–245]. Neuronal progenitors knocked down for LIS1 failed to proliferate [108]. In addition, mosaic analysis demonstrated the requirement of LIS1 for the proliferation of all neuronal lineages and astrocytes [246]. At the initiation of mitosis, *Lis*1^+/−^ neuroblasts exhibited impaired prophase nuclear envelope invagination. This process, which occurs at prophase, is dynein-dependent and facilitates nuclear envelope breakdown [245]. Abnormal interkinetic motility was observed in knockdown, knockout, and increased dosage of LIS1 [108, 155, 241]. LIS1 was found to be essential for precise control of mitotic spindle orientation in both neuroepithelial stem cells and radial glial progenitor cells [247]. Conditional gene knockout of *Lis1*, specifically in neuroepithelial stem cells, resulted in rapid motility of the spindle followed by cell death. Radial glial progenitors were somewhat less affected [247]. Proliferating cells in the ventricular zone with increased LIS1 dosage lost most of their polarity markers and exhibited abnormal adherens junctions [155]. LIS1 genetically interacts with Nde1 in proliferating cells in the ventricular zone; mice with an allelic series of *Lis1* and *Nde1* double mutations displayed a striking dose-dependent size reduction and delamination of the cerebral cortex [248].

DCX participates in the regulation of proliferating neurons in the developing brain in coordination with LIS1. *Dcx* ^−/−^ radial glia cells displayed spindle orientation abnormalities similar to *Lis*1^+/−^ cells that in turn lead to moderate proliferation defects both *in vivo* and *in vitro*. Thus, a functional genetic interaction of the two genes has been demonstrated *in vivo,* where the combined effects of *Lis1* haploinsufficiency and *Dcx* knockout leading to more severe neuronal migration and proliferation phenotypes compared with the single mutants, resulting in cortical disorganization and depletion of the progenitor pool [241]. These results were also confirmed when gene expression was examined. Differential expression analysis indicated that LIS1 and DCX mutants at E14 displayed a repression for cell-cycle processes and networks, while in wild-type embryos these processes are activated [240].

### 7.2. Neuronal Migration in the Developing Brain

Multiple evidence link LIS1 to the regulation of neuronal migration in the developing brain. Abnormal radial migration was noted in an hypomorph allele of *Lis1 (Lis1/sLis1) *[249], in *Lis*1^−/+^ [250], as well as with further reduction of LIS1 using a floxed allele [250], or by knockdown of the gene using *in utero* electroporation [107, 108, 251]. Mosaic analysis also led to the conclusion that LIS1 regulates the migration efficiency in a cell-autonomous manner [246]. In migrating neurons with reduced LIS1 levels, the centrosome and the nucleus were less tightly coupled [223, 251]. *Lis1 *shRNA inhibited somal movement but not process extension [107]. In radially migrating granule cells in mouse cerebellar slices, LIS1 inhibition resulted in slightly different effects; it specifically blocked nuclear migration without affecting the coupling of the centrosome and microtubules in the leading process [252]. LIS1 also affects the migration of inhibitory neurons. It was demonstrated that LIS1 is required for proper tangential migration in *Lis*1^−/+^ [253] and also in case of increased LIS1 dosage [155]. The effects of LIS1 mutation are not limited to the cortex. Abnormal tangential migration was noted in a subset of neurons in the spinal cord [254].

The neuronal migration phenotype was not so obvious in case of *Dcx* mutant mice [255]. Mutant mice showed neocortical lamination that was largely indistinguishable from wild type. Nevertheless, the hippocampus of both heterozygous females and hemizygous males shows disrupted lamination that is most severe in the CA3 region. It has been speculated that the relatively mild phenotype may be due to genetic compensation (review [256]). Supporting this notion, knockdown of *Dcx *using *in utero* electroporation inhibited migration of cortical excitatory neurons both in rat and in mouse brains [4, 54]. Using the same system, it was also possible to demonstrate the genetic interactions between DCX and one of its phosphorylating kinases, MARK2/Par-1 [110]. It is postulated that whereas in case of the knockout there is plenty of time for developmental redundancy mechanisms to become operative, the knockdown involves an acute gene reduction, which may not allow sufficient time for redundancy mechanisms to evolve. This notion received additional support following findings that the knockout of *Dclk1* did not result in an observable phenotype in the migration of pyramidal neurons in the developing brain [39, 55]. Similar to *Dcx*, the knockdown of *Dclk1* impaired the migration of pyramidal neurons [55]. Nevertheless, the double knockout of *Dcx *and *Dclk1 *had a clear effect on cortical development. More specifically, the double mutant mice demonstrated perinatal lethality, disorganized neocortical layering, and profound cytoarchitectural defects of the hippocampus caused by the disruption of radial neuronal migration. The possibility of gene redundancy was investigated in *Dcx* mutant mice, where the expression of transcripts and proteins, which are products of the *Dclk1 *and *Dclk2* gene, was analyzed [46]. A minor change in the expression of one of the DCLK1 proteins was detected in this study. In addition, more severe phenotypes were noted in the combination of mutant alleles for *Dcx *and *Dclk2* [47]. In particular, in the absence of *Dcx* and *Dclk2*, there was a dosage-dependent phenotype in the hippocampus, where hippocampal lamination was disrupted and it was accompanied with simplification of pyramidal dendritic arborizations. Studies in primary hippocampal neuronsrevealed that DCX supported the development of dendritic arbors [237].

*Dcx *knockout had a more pronounced effect on the migration of interneurons. Branching and nucleokinesis problems were observed in interneurons derived from *Dcx* mutant mouse brains [257]. *In utero* electroporation of *Dcx* shRNA impaired tangential migration [258]. In addition, these type of experiments also indicated the LIS1 and DCX work in the same genetic pathway in inhibitory neurons migrating through the lateral cortical stream, which supplies neurons to structures in the ventral telencephalon including the amygdala and piriform cortex [259]. DCX also plays an important role in migration of neurons in the adult mouse to the olfactory bulb using the rostral migratory stream [56]. *Dcx* RNAi reduced SVZ cell migration *in vitro*, both cell autonomously and noncell autonomously [260].

### 7.3. Postnatal Effects

The effects of LIS1 mutations are not confined to embryonic stages. *Lis1* mutant mice develop spontaneous seizures and enhanced excitation [261]. Several abnormalities were noted in the hippocampus: abnormal inhibitory inputs [262] and dysfunctional synaptic integration of granule cells generated in the developing and adult dentate gyrus [263]. In addition, postnatally the *Lis1/sLis1* mutant mouse exhibited alterations of the inhibitory synaptic responses recorded from cortical pyramidal neurons [264]. LIS1 has been shown to be critical for determining the synaptic distribution on interneuron dendrites [265]. Therefore, it is possible to speculate that in addition to migration deficits, LIS1-dependent regulation of synaptic mobility may promote epilepsy by disrupting excitatory inputs onto GABAergic interneurons.

The lamination defects in the hippocampus of *Dcx *mutant mice result in the development of epilepsy in these animals [266–268]. Furthermore, also *in utero *electroporation of *Dcx *shRNA revealed abnormalities in the hippocampal network [269] and spontaneous epileptic seizures [270].

### 7.4. Possible Reversal of the Developmental Phenotype

LIS1 protein degradation was shown to be mediated, at least in part, by the protein protease, calpain [271]. Therefore, the possibility that interference with this pathway may relieve at least part of the phenotype was tested [271, 272] (review [273]). Treatment with calpain inhibitors or knockdown of calpain expression by siRNA improved the abnormal cellular phenotype caused by heterozygous loss of *Lis1 *in cell culture. Calpain inhibitors were also administered to pregnant *Lis*1^+/−^ mice during embryonic corticogenesis. Remarkably, both inhibition by drugs or knockdown of calpain *in vivo *rescued defective neuronal migration and abnormal cortical and hippocampal layering in heterozygous mutant pups born to treated mothers. Furthermore, these mutant pups also exhibited improved motor function. The therapeutic potential of a calpain inhibitor was also tested on postnatal lissencephalic cells [274]. Interestingly, application of the calpain inhibitor restored spontaneous and miniature EPSC (excitatory postsynaptic current) frequencies to wild-type levels without affecting inhibitory postsynaptic synaptic current. However, western blot analysis of protein expression, including proteins involved in excitatory synaptic transmission, demonstrated that the cleavage of the calpain substrate alpha II-spectrin was blocked, but the levels of the LIS1 protein were not restored, suggesting the possibility that the rescue was through an indirect route, which did not involve LIS1 levels.

Delayed reexpression of *Dcx *in already formed SBH after birth led to SBH regression [275] (review [273]). Moreover, the reexpression of *Dcx* corrected the sensitivity to convulsant-inducing drugs. Reduction of the SBH demonstrated a restricted time window and was limited to early postnatal ages. An additional avenue, which was not investigated at the postnatal stage, was the demonstrated genetic interaction between DCX and Par-1/MARK2. The combined knockdown of Par-1/MARK2 and DCX significantly rescued the neuronal migration deficit and the subcellular processivity of centrosomal motility [110]. Therefore, the above-mentioned studies raise the possibility that neuronal migration disorders may be eventually treatable by molecular or pharmacological interventions.

## References

[1] Cajal S (1995). Neurons: size and general morphology. *Histology of the Nervous System*.

[2] Ayala R, Shu T, Tsai LH (2007). Trekking across the brain: the journey of neuronal migration. *Cell*.

[3] Hatten ME (2002). New directions in neuronal migration. *Science*.

[4] Kriegstein AR, Noctor SC (2004). Patterns of neuronal migration in the embryonic cortex. *Trends in Neurosciences*.

[5] Lambert de Rouvroit C, Goffinet AM (2001). Neuronal migration. *Mechanisms of Development*.

[6] Nadarajah B, Parnavelas JG (2002). Modes of neuronal migration in the developing cerebral cortex. *Nature Reviews Neuroscience*.

[7] Jaglin XH, Chelly J (2009). Tubulin-related cortical dysgeneses: microtubule dysfunction underlying neuronal migration defects. *Trends in Genetics*.

[8] Dobyns WB, Andermann E, Andermann F (1996). X-linked malformations of neuronal migration. *Neurology*.

[9] Fox JW, Walsh CA (1999). Periventricular heterotopia and the genetics of neuronal migration in the cerebral cortex. *American Journal of Human Genetics*.

[10] Guerrini R, Filippi T (2005). Neuronal migration disorders, genetics, and epileptogenesis. *Journal of Child Neurology*.

[11] Reiner O, Cahana A, Shmueli O, Leeor A, Sapir T (1996). LISI, a gene involved in a neuronal migration disorder: from gene isolation towards analysis of gene function. *Cellular Pharmacology*.

[12] Ross ME, Walsh CA (2001). Human brain malformations and their lessons for neuronal migration. *Annual Review of Neuroscience*.

[13] Walsh CA (2000). Genetics of neuronal migration in the cerebral cortex. *Mental Retardation and Developmental Disabilities Research Reviews*.

[14] Farrell MA, DeRosa MJ, Curran JG (1992). Neuropathologic findings in cortical resections (including hemispherectomies) performed for the treatment of intractable childhood epilepsy. *Acta Neuropathologica*.

[15] Aicardi J (1994). The place of neuronal migration abnormalities in child neurology. *Canadian Journal of Neurological Sciences*.

[16] Vaillend C, Poirier R, Laroche S (2008). Genes, plasticity and mental retardation. *Behavioural Brain Research*.

[17] Rakic P, Hashimoto-Torii K, Sarkisian MR (2007). Genetic determinants of neuronal migration in the cerebral cortex. *Novartis Foundation Symposium*.

[18] Kuijpers M, Hoogenraad CC (2011). Centrosomes, microtubules and neuronal development. *Molecular and Cellular Neuroscience*.

[19] Fatemi SH, Folsom TD (2009). The neurodevelopmental hypothesis of Schizophrenia, revisited. *Schizophrenia Bulletin*.

[20] Bunney BG, Potkin SG, Bunney WE (1995). New morphological and neuropathological findings in schizophrenia: a neurodevelopmental perspective. *Clinical Neuroscience*.

[21] Peterson BS (1995). Neuroimaging in child and adolescent neuropsychiatric disorders. *Journal of the American Academy of Child and Adolescent Psychiatry*.

[22] Götz M, Huttner WB (2005). The cell biology of neurogenesis. *Nature Reviews Molecular Cell Biology*.

[23] Hevner RF (2006). From radial glia to pyramidal-projection neuron: transcription factor cascades in cerebral cortex development. *Molecular Neurobiology*.

[24] Shitamukai A, Matsuzaki F (2012). Control of asymmetric cell division of mammalian neural progenitors. *Development, Growth & Differentiation*.

[25] Lehtinen MK, Walsh CA (2011). Neurogenesis at the brain-cerebrospinal fluid interface. *Annual Review of Cell and Developmental Biology*.

[26] Fietz SA, Huttner WB (2011). Cortical progenitor expansion, self-renewal and neurogenesis-a polarized perspective. *Current Opinion in Neurobiology*.

[27] Gal JS, Morozov YM, Ayoub AE, Chatterjee M, Rakic P, Haydar TF (2006). Molecular and morphological heterogeneity of neural precursors in the mouse neocortical proliferative zones. *Journal of Neuroscience*.

[28] Stancik EK, Navarro-Quiroga I, Sellke R, Haydar TF (2010). Heterogeneity in ventricular zone neural precursors contributes to neuronal fate diversity in the postnatal neocortex. *Journal of Neuroscience*.

[29] Noctor SC, Flint AC, Weissman TA, Dammerman RS, Kriegstein AR (2001). Neurons derived from radial glial cells establish radial units in neocortex. *Nature*.

[30] Miyata T, Kawaguchi A, Okano H, Ogawa M (2001). Asymmetric inheritance of radial glial fibers by cortical neurons. *Neuron*.

[31] Malatesta P, Hartfuss E, Götz M (2000). Isolation of radial glial cells by fluorescent-activated cell sorting reveals a neural lineage. *Development*.

[32] Anthony TE, Klein C, Fishell G, Heintz N (2004). Radial glia serve as neuronal progenitors in all regions of the central nervous system. *Neuron*.

[33] Frade JM (2002). Interkinetic nuclear movement in the vertebrate neuroepithelium: encounters with an old acquaintance. *Progress in Brain Research*.

[34] Baye LM, Link BA (2007). Interkinetic nuclear migration and the selection of neurogenic cell divisions during vertebrate retinogenesis. *Journal of Neuroscience*.

[35] Reiner O, Sapir T, Gerlitz G (2012). Interkinetic nuclear movement in the ventricular zone of the cortex. *Journal of Molecular Neuroscience*.

[36] Kosodo Y, Röper K, Haubensak W, Marzesco AM, Corbeil D, Huttner WB (2004). Asymmetric distribution of the apical plasma membrane during neurogenic divisions of mamalian neuroepithelial cells. *EMBO Journal*.

[37] Noctor SC, Martínez-Cerdeño V, Kriegstein AR (2008). Distinct behaviors of neural stem and progenitor cells underlie cortical neurogenesis. *Journal of Comparative Neurology*.

[38] Konno D, Shioi G, Shitamukai A (2008). Neuroepithelial progenitors undergo LGN-dependent planar divisions to maintain self-renewability during mammalian neurogenesis. *Nature Cell Biology*.

[39] Alexandre P, Reugels AM, Barker D, Blanc E, Clarke JDW (2010). Neurons derive from the more apical daughter in asymmetric divisions in the zebrafish neural tube. *Nature Neuroscience*.

[40] Shitamukai A, Konno D, Matsuzaki F (2011). Oblique radial glial divisions in the developing mouse neocortex induce self-renewing progenitors outside the germinal zone that resemble primate outer subventricular zone progenitors. *Journal of Neuroscience*.

[41] Reugels AM, Boggetti B, Scheer N, Campos-Ortega JA (2006). Asymmetric localization of numb: EGFP in dividing neuroepithelial cells during neurulation in Danio rerio. *Developmental Dynamics*.

[42] Wakamatsu Y, Nakamura N, Lee JA, Cole GJ, Osumi N (2007). Transitin, a nestin-like intermediate filament protein, mediates cortical localization and the lateral transport of Numb in mitotic avian neuroepithelial cells. *Development*.

[43] Noctor SC, Martinez-Cerdeño V, Ivic L, Kriegstein AR (2004). Cortical neurons arise in symmetric and asymmetric division zones and migrate through specific phases. *Nature Neuroscience*.

[44] Takahashi T, Nowakowski RS, Caviness VS (1995). The cell cycle of the pseudostratified ventricular epithelium of the embryonic murine cerebral wall. *Journal of Neuroscience*.

[45] Calegari F, Haubensak W, Haffher C, Huttner WB (2005). Selective lengthening of the cell cycle in the neurogenic subpopulation of neural progenitor cells during mouse brain development. *Journal of Neuroscience*.

[46] Calegari F, Huttner WB (2003). An inhibition of cyclin-dependent kinases that lengthens, but does not arrest, neuroepithelial cell cycle induces premature neurogenesis. *Journal of Cell Science*.

[47] Haubensak W, Attardo A, Denk W, Huttner WB (2004). Neurons arise in the basal neuroepithelium of the early mammalian telencephalon: a major site of neurogenesis. *Proceedings of the National Academy of Sciences of the United States of America*.

[48] Miyata T, Kawaguchi A, Saito K, Kawano M, Muto T, Ogawa M (2004). Asymmetric production of surface-dividing and non-surface-dividing cortical progenitor cells. *Development*.

[49] Smart IHM (1973). Proliferative characteristics of the ependymal layer during the early development of the mouse neocortex: a pilot study based on recording the number, location and plane of cleavage of mitotic figures. *Journal of Anatomy*.

[50] Tabata H, Yoshinaga S, Nakajima K (2012). Cytoarchitecture of mouse and human subventricular zone in developing cerebral neocortex. *Experimental Brain Research Experimentelle Hirnforschung Experimentation Cerebrale*.

[51] Takahashi T, Nowakowski RS, Caviness VS (1993). Cell cycle parameters and patterns of nuclear movement in the neocortical proliferative zone of the fetal mouse. *Journal of Neuroscience*.

[52] Carney RSE, Bystron I, López-Bendito G, Molnár Z (2007). Comparative analysis of extra-ventricular mitoses at early stages of cortical development in rat and human. *Brain Structure and Function*.

[53] Smart IHM, Dehay C, Giroud P, Berland M, Kennedy H (2002). Unique morphological features of the proliferative zones and postmitotic compartments of the neural epithelium giving rise to striate and extrastriate cortex in the monkey. *Cerebral Cortex*.

[54] Zecevic N, Chen Y, Filipovic R (2005). Contributions of cortical subventricular zone to the development of the human cerebral cortex. *Journal of Comparative Neurology*.

[55] Fish JL, Dehay C, Kennedy H, Huttner WB (2008). Making bigger brains—the evolution of neural-progenitor-cell division. *Journal of Cell Science*.

[56] Rakic P (1974). Neurons in rhesus monkey visual cortex: systematic relation between time of origin and eventual disposition. *Science*.

[57] Lukaszewicz A, Savatier P, Cortay V (2005). G1 phase regulation, area-specific cell cycle control, and cytoarchitectonics in the primate cortex. *Neuron*.

[58] Hansen DV, Lui JH, Parker PRL, Kriegstein AR (2010). Neurogenic radial glia in the outer subventricular zone of human neocortex. *Nature*.

[59] Fietz SA, Kelava I, Vogt J (2010). OSVZ progenitors of human and ferret neocortex are epithelial-like and expand by integrin signaling. *Nature Neuroscience*.

[60] Wang X, Tsai JW, Lamonica B, Kriegstein AR (2011). A new subtype of progenitor cell in the mouse embryonic neocortex. *Nature Neuroscience*.

[61] Anderson SA, Eisenstat DD, Shi L, Rubenstein JLR (1997). Interneuron migration from basal forebrain to neocortex: dependence on Dlx genes. *Science*.

[62] Wonders C, Anderson SA (2005). Cortical interneurons and their origins. *Neuroscientist*.

[63] Gelman DM, Marín O (2010). Generation of interneuron diversity in the mouse cerebral cortex. *European Journal of Neuroscience*.

[64] Wonders CP, Anderson SA (2006). The origin and specification of cortical interneurons. *Nature Reviews Neuroscience*.

[65] Xu Q, Cobos I, De La Cruz ED, Rubenstein JL, Anderson SA (2004). Origins of cortical interneuron subtypes. *Journal of Neuroscience*.

[66] Jiménez D, López-Mascaraque LM, Valverde F, De Carlos JA (2002). Tangential migration in neocortical development. *Developmental Biology*.

[67] Taglialatela P, Soria JM, Caironi V, Moiana A, Bertuzzi S (2004). Compromised generation of GABAergic interneurons in the brains of Vax1-/- mice. *Development*.

[68] Gelman DM, Martini FJ, Nóbrega-Pereira S, Pierani A, Kessaris N, Marín O (2009). The embryonic preoptic area is a novel source of cortical GABAergic interneurons. *Journal of Neuroscience*.

[69] Tan X, Shi SH (2012). Neocortical neurogenesis and neuronal migration. *Wiley Interdisciplinary Reviews*.

[70] Mori T, Buffo A, Götz M (2005). The novel roles of glial cells revisited: the contribution of radial glia and astrocytes to neurogenesis. *Current Topics in Developmental Biology*.

[71] Campbell K, Götz M (2002). Radial glia: multi-purpose cells for vertebrate brain development. *Trends in Neurosciences*.

[72] Brown KN, Chen S, Han Z (2011). Clonal production and organization of inhibitory interneurons in the neocortex. *Science*.

[73] Yun K, Potter S, Rubenstein JLR (2001). Gsh2 and Pax6 play complementary roles in dorsoventral patterning of the mammalian telencephalon. *Development*.

[74] Englund C, Fink A, Lau C (2005). Pax6, Tbr2, and Tbr1 are expressed sequentially by radial glia, intermediate progenitor cells, and postmitotic neurons in developing neocortex. *Journal of Neuroscience*.

[75] Muzio L, DiBenedetto B, Stoykova A, Boncinelli E, Gruss P, Mallamaci A (2002). Conversion of cerebral cortex into basal ganglia in Emx2-/- Pax6Sey/Sey double-mutant mice. *Nature Neuroscience*.

[76] Malatesta P, Hack MA, Hartfuss E (2003). Neuronal or glial progeny: regional differences in radial glia fate. *Neuron*.

[77] Toresson H, Potter SS, Campbell K (2000). Genetic control of dorsal-ventral identity in the telencephalon: opposing roles for Pax6 and Gsh2. *Development*.

[78] Sheth AN, Bhide PG (1997). Concurrent cellular output from two proliferative populations in the early embryonic mouse corpus striatum. *The Journal of Comparative Neurology*.

[79] Marín O, Rubenstein JLR (2003). Cell migration in the forebrain. *Annual Review of Neuroscience*.

[80] Marín O, Rubenstein JLR (2001). A long, remarkable journey: tangential migration in the telencephalon. *Nature Reviews Neuroscience*.

[81] Tsai LH, Gleeson JG (2005). Nucleokinesis in neuronal migration. *Neuron*.

[82] Reiner O, Gerlitz G, Rubinstein JR, Marin O (2013). Nucleokinesis. *Developmental Neuroscience: A Comprehensive Reference*.

[83] Angevine JB, Sidman RL (1961). Autoradiographic study of cell migration during histogenesis of cerebral cortex in the mouse. *Nature*.

[84] McConnell SK (1991). The generation of neuronal diversity in the central nervous system. *Annual Review of Neuroscience*.

[85] Nery S, Fishell G, Corbin JG (2002). The caudal ganglionic eminence is a source of distinct cortical and subcortical cell populations. *Nature Neuroscience*.

[86] Miller MW (1985). Cogeneration of retrogradely labeled corticocortical projection and GABA-immunoreactive local circuit neurons in cerebral cortex. *Brain Research*.

[87] Fairen A, Cobas A, Fonseca M (1986). Times of generation of glutamic acid decarboxylase immunoreactive neurons in mouse somatosensory cortex. *Journal of Comparative Neurology*.

[88] Valcanis H, Tan SS (2003). Layer specification of transplanted interneurons in developing mouse neocortex. *The Journal of Neuroscience*.

[89] Rakic P (1972). Mode of cell migration to the superficial layers of fetal monkey neocortex. *Journal of Comparative Neurology*.

[90] Hatten ME (1999). Central nervous system neuronal migration. *Annual Review of Neuroscience*.

[91] Lavdas AA, Grigoriou M, Pachnis V, Parnavelas JG (1999). The medial ganglionic eminence gives rise to a population of early neurons in the developing cerebral cortex. *The Journal of Neuroscience*.

[92] De Carlos JA, López-Mascaraque L, Valverde F (1996). Dynamics of cell migration from the lateral ganglionic eminence in the rat. *The Journal of Neuroscience*.

[93] Tan SS, Kalloniatis M, Sturm K, Tam PPL, Reese BE, Faulkner-Jones B (1998). Separate progenitors for radial and tangential cell dispersion during development of the cerebral neocortex. *Neuron*.

[94] Ware ML, Tavazoie SF, Reid CB, Walsh CA (1999). Coexistence of widespread clones and large radial clones in early embryonic ferret cortex. *Cerebral Cortex*.

[95] Anderson S, Mione M, Yun K, Rubenstein JLR (1999). Differential origins of neocortical projection and local circuit neurons: role of Dlx genes in neocortical interneuronogenesis. *Cerebral Cortex*.

[96] Anderson SA, Marín O, Horn C, Jennings K, Rubenstein JLR (2001). Distinct cortical migrations from the medial and lateral ganglionic eminences. *Development*.

[97] Polleux F, Whitford KL, Dijkhuizen PA, Vitalis T, Ghosh A (2002). Control of cortical interneuron migration by neurotrophins and PI3-kinase signaling. *Development*.

[98] Wichterle H, Turnbull DH, Nery S, Fishell G, Alvarez-Buylla A (2001). In utero fate mapping reveals distinct migratory pathways and fates of neurons born in the mammalian basal forebrain. *Development*.

[99] Yozu M, Tabata H, Nakajima K (2005). The Caudal migratory stream: a novel migratory stream of interneurons derived from the caudal ganglionic eminence in the developing mouse forebrain. *The Journal of Neuroscience*.

[100] Miyoshi G, Hjerling-Leffler J, Karayannis T (2010). Genetic fate mapping reveals that the caudal ganglionic eminence produces a large and diverse population of superficial cortical interneurons. *The Journal of Neuroscience*.

[101] Ochiai W, Minobe S, Ogawa M, Miyata T (2007). Transformation of pin-like ventricular zone cells into cortical neurons. *Neuroscience Research*.

[102] Tabata H, Nakajima K (2003). Multipolar migration: the third mode of radial neuronal migration in the developing cerebral cortex. *The Journal of Neuroscience*.

[103] Noctor SC, Flint AC, Weissman TA, Wong WS, Clinton BK, Kriegstein AR (2002). Dividing precursor cells of the embryonic cortical ventricular zone have morphological and molecular characteristics of radial glia. *The Journal of Neuroscience*.

[104] LoTurco JJ, Bai J (2006). The multipolar stage and disruptions in neuronal migration. *Trends in Neurosciences*.

[105] Reiner O, Sapir T (2009). Polarity regulation in migrating neurons in the cortex. *Molecular Neurobiology*.

[106] Sakakibara A, Sato T, Ando R, Noguchi N, Masaoka M, Miyata T (2013). Dynamics of centrosome translocation and microtubule organization in neocortical neurons during distinct modes of polarization. *Cerebral Cortex*.

[107] Tsai JW, Bremner KH, Vallee RB (2007). Dual subcellular roles for LIS1 and dynein in radial neuronal migration in live brain tissue. *Nature Neuroscience*.

[108] Tsai JW, Chen Y, Kriegstein AR, Vallee RB (2005). LIS1 RNA interference blocks neural stem cell division, morphogenesis, and motility at multiple stages. *Journal of Cell Biology*.

[109] Sapir T, Sapoznik S, Levy T (2008). Accurate balance of the polarity kinase MARK2/Par-1 is required for proper cortical neuronal migration. *The Journal of Neuroscience*.

[110] Sapir T, Shmueli A, Levy T (2008). Antagonistic effects of doublecortin and MARK2/Par-1 in the developing cerebral cortex. *The Journal of Neuroscience*.

[111] Horio T, Hotani H (1986). Visualization of the dynamic instability of individual microtubules by dark-field microscopy. *Nature*.

[112] Mitchinson T, Kirschner M (1984). Dynamic instability of microtubule growth. *Nature*.

[113] Sammak PJ, Borisy GG (1988). Direct observation of microtubule dynamics in living cells. *Nature*.

[114] Schulze E, Kirschner M (1988). New features of microtubule behaviour observed in vivo. *Nature*.

[115] Cassimeris L, Pryer NK, Salmon ED (1988). Real-time observations of microtubule dynamic instability in living cells. *Journal of Cell Biology*.

[116] Baas PW (1999). Microtubules and neuronal polarity: lessons from mitosis. *Neuron*.

[117] Stiess M, Bradke F (2011). Neuronal polarization: the cytoskeleton leads the way. *Developmental Neurobiology*.

[118] Higginbotham HR, Gleeson JG (2007). The centrosome in neuronal development. *Trends in Neurosciences*.

[119] De Anda FC, Pollarolo G, Da Silva JS, Camoletto PG, Feiguin F, Dotti CG (2005). Centrosome localization determines neuronal polarity. *Nature*.

[120] De Anda FC, Meletis K, Ge X, Rei D, Tsai LH (2010). Centrosome motility is essential for initial axon formation in the neocortex. *The Journal of Neuroscience*.

[121] Distel M, Hocking JC, Volkmann K, Köster RW (2010). The centrosome neither persistently leads migration nor determines the site of axonogenesis in migrating neurons in vivo. *Journal of Cell Biology*.

[122] Gartner A, Fornasiero EF, Munck S (2012). N-cadherin specifies first asymmetry in developing neurons. *The EMBO Journal*.

[123] Falnikar A, Baas PW (2009). Critical roles for microtubules in axonal development and disease. *Results and Problems in Cell Differentiation*.

[124] Stiess M, Maghelli N, Kapitein LC (2010). Axon extension occurs independently of centrosomal microtubule nucleation. *Science*.

[125] Baas PW, Deitch JS, Black MM, Banker GA (1988). Polarity orientation of microtubules in hippocampal neurons: uniformity in the axon and nonuniformity in the dendrite. *Proceedings of the National Academy of Sciences of the United States of America*.

[126] Stepanova T, Slemmer J, Hoogenraad CC (2003). Visualization of microtubule growth in cultured neurons via the use of EB3-GFP (end-binding protein 3-green fluorescent protein). *The Journal of Neuroscience*.

[127] Reiner O (1999). The unfolding story of two lissencephaly genes and brain development. *Molecular Neurobiology*.

[128] Barkovich AJ, Kuzniecky RI, Dobyns WB, Jackson GD, Becker LE, Evrard P (1996). A classification scheme for malformations of cortical development. *Neuropediatrics*.

[129] Kurlemann G, Schuierer G, Kuchelmeister K, Kleine M, Weglage J, Palm DG (1993). Lissencephaly syndromes: clinical aspects. *Child’s Nervous System*.

[130] Pancoast M, Dobyns W, Golden JA (2005). Interneuron deficits in patients with the Miller-Dieker syndrome. *Acta Neuropathologica*.

[131] Shimojima K, Sugiura C, Takahashi H (2010). Genomic copy number variations at 17p13.3 and epileptogenesis. *Epilepsy Research*.

[132] Reiner O, Carrozzo R, Shen Y (1993). Isolation of a Miller-Dieker lissencephaly gene containing G protein *β*- subunit-like repeats. *Nature*.

[133] Des Portes V, Pinard JM, Billuart P (1998). A novel CNS gene required for neuronal migration and involved in X- linked subcortical laminar heterotopia and lissencephaly syndrome. *Cell*.

[134] Gleeson JG, Allen KM, Fox JW (1998). doublecortin, a brain-specific gene mutated in human X-linked lissencephaly and double cortex syndrome, encodes a putative signaling protein. *Cell*.

[135] Keays DA, Tian G, Poirier K (2007). Mutations in *α*-tubulin cause abnormal neuronal migration in mice and lissencephaly in humans. *Cell*.

[136] Poirier K, Keays DA, Francis F (2007). Large spectrum of lissencephaly and pachygyria phenotypes resulting from de novo missense mutations in tubulin alpha 1A (TUBA1A). *Human Mutation*.

[137] Mokanszki A, Korhegyi I, Szabo N (2012). Lissencephaly and band heterotopia: LIS1, TUBA1A, and DCX mutations in hungary. *Journal of Child Neurology*.

[138] Dobyns WB, Reiner O, Carrozzo R, Ledbetter DH (1993). Lissencephaly: a human brain malformation associated with deletion of the LIS1 gene located at chromosome 17p13. *Journal of the American Medical Association*.

[139] Pilz DT, Matsumoto N, Minnerath S (1998). LIS1 and XLIS (DCX) mutations cause most classical lissencephaly, but different patterns of malformation. *Human Molecular Genetics*.

[140] Dobyns WB, Truwit CL, Ross ME (1999). Differences in the gyral pattern distinguish chromosome 17-linked and X-linked lissencephaly. *Neurology*.

[141] Viot G, Sonigo P, Simon I (2004). Neocortical neuronal arrangement in LIS1 and DCX lissencephaly may be different. *American Journal of Medical Genetics*.

[142] Sicca F, Kelemen A, Genton P (2003). Mosaic mutations of the LIS1 gene cause subcortical band heterotopia. *Neurology*.

[143] Mineyko A, Doja A, Hurteau J, Dobyns WB, Das S, Boycott KM (2010). A novel missense mutation in LIS1 in a child with subcortical band heterotopia and pachygyria inherited from his mildly affected mother with somatic mosaicism. *Journal of Child Neurology*.

[144] Jaglin XH, Poirier K, Saillour Y (2009). Mutations in the *β*-tubulin gene TUBB2B result in asymmetrical polymicrogyria. *Nature Genetics*.

[145] Guerrini R, Dobyns WB, Barkovich AJ (2008). Abnormal development of the human cerebral cortex: genetics, functional consequences and treatment options. *Trends in Neurosciences*.

[146] Lockrow JP, Holden KR, Dwivedi A, Matheus MG, Lyons MJ (2012). LIS1 duplication: expanding the phenotype. *Journal of Child Neurology*.

[147] Harding B, Guerrini R, Andermann F, Canapicchi R, Roger J, Zilfkin B, Pfanner P (1996). Gray matter heterotopia. *Dysplasias of Cerebral Cortex and Epilepsy*.

[148] Weinberger DR (1987). Implications of normal brain development for the pathogenesis of schizophrenia. *Archives of General Psychiatry*.

[149] Reiner O, Sapoznik S, Sapir T (2006). Lissencephaly 1 linking to multiple diseases: mental retardation, neurodegeneration, schizophrenia, male sterility, and more. *NeuroMolecular Medicine*.

[150] Geschwind DH, Levitt P (2007). Autism spectrum disorders: developmental disconnection syndromes. *Current Opinion in Neurobiology*.

[151] Maussion G, Carayol J, Lepagnol-bestel AM (2008). Convergent evidence identifying MAP/microtubule affinity-regulating kinase 1 (MARK1) as a susceptibility gene for autism. *Human Molecular Genetics*.

[152] Tassan JP, Le Goff X (2004). An overview of the KIN1/PAR-1/MARK kinase family. *Biology of the Cell*.

[153] Drewes G, Ebneth A, Preuss U, Mandelkow EM, Mandelkow E (1997). MARK, a novel family of protein kinases that phosphorylate microtubule- associated proteins and trigger microtubule disruption. *Cell*.

[154] Drewes G, Trinczek B, Illenberger S (1995). Microtubule-associated protein/microtubule affinity-regulating kinase (p110^mark^). A novel protein kinase that regulates tau-microtubule interactions and dynamic instability by phosphorylation at the Alzheimer-specific site serine 262. *Journal of Biological Chemistry*.

[155] Bi W, Sapir T, Shchelochkov OA (2009). Increased LIS1 expression affects human and mouse brain development. *Nature Genetics*.

[156] Avela K, Aktan-Collan K, Horelli-Kuitunen N, Knuutila S, Somer M (2011). A microduplication on chromosome 17p13.1p13.3 including the PAFAH1B1 (LIS1) gene. *American Journal of Medical Genetics A*.

[157] Bruno DL, Anderlid BM, Lindstrand A (2010). Further molecular and clinical delineation of co-locating 17p13.3 microdeletions and microduplications that show distinctive phenotypes. *Journal of Medical Genetics*.

[158] Shaw-Smith C, Pittman AM, Willatt L (2006). Microdeletion encompassing MAPT at chromosome 17q21.3 is associated with developmental delay and learning disability. *Nature Genetics*.

[159] Sharp AJ, Hansen S, Selzer RR (2006). Discovery of previously unidentified genomic disorders from the duplication architecture of the human genome. *Nature Genetics*.

[160] Koolen DA, Vissers LELM, Pfundt R (2006). A new chromosome 17q21.31 microdeletion syndrome associated with a common inversion polymorphism. *Nature Genetics*.

[161] Varela MC, Krepischi-Santos ACV, Paz JA (2006). A 17q21.31 microdeletion encompassing the MAPT gene in a mentally impaired patient. *Cytogenetic and Genome Research*.

[162] Koolen DA, Sharp AJ, Hurst JA (2008). Clinical and molecular delineation of the 17q21.31 microdeletion syndrome. *Journal of Medical Genetics*.

[163] Bullmann T, Holzer M, Mori H, Arendt T (2009). Pattern of tau isoforms expression during development in vivo. *International Journal of Developmental Neuroscience*.

[164] Takuma H, Arawaka S, Mori H (2003). Isoforms changes of tau protein during development in various species. *Developmental Brain Research*.

[165] Weingarten MD, Lockwood AH, Hwo SY, Kirschner MW (1975). A protein factor essential for microtubule assembly. *Proceedings of the National Academy of Sciences of the United States of America*.

[166] Cleveland DW, Hwo SY, Kirschner MW (1977). Purification of tau, a microtubule associated protein that induces assembly of microtubules from purified tubulin. *Journal of Molecular Biology*.

[167] Reiner O, Shmueli A, Sapir T (2009). Neuronal migration and neurodegeneration: 2 sides of the same coin. *Cerebral Cortex*.

[168] Billingsley ML, Kincaid RL (1997). Regulated phosphorylation and dephosphorylation of tau protein: effects on microtubule interaction, intracellular trafficking and neurodegeneration. *Biochemical Journal*.

[169] Ballatore C, Lee VMY, Trojanowski JQ (2007). Tau-mediated neurodegeneration in Alzheimer’s disease and related disorders. *Nature Reviews Neuroscience*.

[170] Dehmelt L, Halpain S (2004). Actin and microtubules in neurite initiation: are maps the missing link?. *Journal of Neurobiology*.

[171] Stokin GB, Goldstein LSB (2006). Axonal transport and Alzheimer’s disease. *Annual Review of Biochemistry*.

[172] Gerdes JM, Katsanis N (2005). Microtubule transport defects in neurological and ciliary disease. *Cellular and Molecular Life Sciences*.

[173] Holzbaur ELF (2004). Motor neurons rely on motor proteins. *Trends in Cell Biology*.

[174] Baas PW, Qiang L (2005). Neuronal microtubules: when the MAP is the roadblock. *Trends in Cell Biology*.

[175] Willins DA, Xiang X, Morris NR (1995). An alpha tubulin mutation suppresses nuclear migration mutations in Aspergillus nidulans. *Genetics*.

[176] Sapir T, Elbaum M, Reiner O (1997). Reduction of microtubule catastrophe events by LIS1, platelet-activating factor acetylhydrolase subunit. *EMBO Journal*.

[177] Smith DS, Niethammer M, Ayala R (2000). Regulation of cytoplasmic dynein behaviour and microtubule organization by mammalian Lis1. *Nature Cell Biology*.

[178] Sasaki S, Shionoya A, Ishida M (2000). A LIS1/NUDEL/cytoplasmic dynein heavy chain complex in the developing and adult nervous system. *Neuron*.

[179] Sumigray KD, Chen H, Lechler T (2011). Lis1 is essential for cortical microtubule organization and desmosome stability in the epidermis. *Journal of Cell Biology*.

[180] Rehberg M, Kleylein-Sohn J, Faix J, Ho TH, Schulz I, Gräf R (2005). Dictyostelium LIS1 is a centrosomal protein required for microtubule/cell cortex interactions, nucleus/centrosome linkage, and actin dynamics. *Molecular Biology of the Cell*.

[181] Sapir T, Cahana A, Seger R, Nekhai S, Reiner O (1999). LIS1 is a microtubule-associated phosphoprotein. *European Journal of Biochemistry*.

[182] Caspi M, Atlas R, Kantor A, Sapir T, Reiner O (2000). Interaction between LIS1 and doublecortin, two lissencephaly gene products. *Human Molecular Genetics*.

[183] Coquelle FM, Caspi M, Cordelières FP (2002). LIS1, CLIP-170’s key to the dynein/dynactin pathway. *Molecular and Cellular Biology*.

[184] Jiménez-Mateos EM, Wandosell F, Reiner O, Avila J, González-Billault C (2005). Binding of microtubule-associated protein 1B to LIS1 affects the interaction between dynein and LIS1. *Biochemical Journal*.

[185] Kholmanskikh SS, Dobrin JS, Wynshaw-Boris A, Letourneau PC, Ross ME (2003). Disregulated RhoGTPases and actin cytoskeleton contribute to the migration defect in Lis1-deficient neurons. *The Journal of Neuroscience*.

[186] Kholmanskikh SS, Koeller HB, Wynshaw-Boris A, Gomez T, Letourneau PC, Ross ME (2006). Calcium-dependent interaction of Lis1 with IQGAP1 and Cdc42 promotes neuronal motility. *Nature Neuroscience*.

[187] Beckwith SM, Roghi CH, Liu B, Morris NR (1998). The ’8-kD’ cytoplasmic dynein light chain is required for nuclear migration and for dynein heavy chain localization in Aspergillus nidulans. *Journal of Cell Biology*.

[188] Xiang X, Beckwith SM, Morris NR (1994). Cytoplasmic dynein is involved in nuclear migration in Aspergillus nidulans. *Proceedings of the National Academy of Sciences of the United States of America*.

[189] Xiang X, Han G, Winkelmann DA, Zuo W, Morris NR (2000). Dynamics of cytoplasmic dynein in living cells and the effect of a mutation in the dynactin complex actin-related protein Arp1. *Current Biology*.

[190] Liu Z, Xie T, Steward R (1999). Lis1, the Drosophila homolog of a human lissencephaly disease gene, is required for germline cell division and oocyte differentiation. *Development*.

[191] Swan A, Nguyen T, Suter B (1999). Drosophila Lissencephaly-1 functions with Bic-D and dynein in oocyte determination and nuclear positioning. *Nature Cell Biology*.

[192] Lei Y, Warrior R (2000). The Drosophila lissencephalyl (DLis1) gene is required for nuclear migration. *Developmental Biology*.

[193] Fujiwara T, Tanaka K, Inoue E, Kikyo M, Takai Y (1999). Bni1p regulates microtubule-dependent nuclear migration through the actin cytoskeleton in Saccharomyces cerevisiae. *Molecular and Cellular Biology*.

[194] Lee WL, Oberle JR, Cooper JA (2003). The role of the lissencephaly protein Pac1 during nuclear migration in budding yeast. *Journal of Cell Biology*.

[195] Faulkner NE, Dujardin DL, Tai CY (2000). A role for the lissencephaly gene Lis1 in mitosis and cytoplasmic dynein function. *Nature Cell Biology*.

[196] Tai CY, Dujardin DL, Faulkner NE, Vallee RB (2002). Role of dynein, dynactin, and CLIP-170 interactions in LIS1 kinetochore function. *Journal of Cell Biology*.

[197] Liu Z, Steward R, Luo L (2000). Drosophila Lis1 is required for neuroblast proliferation, dendritic elaboration and axonal transport. *Nature Cell Biology*.

[198] Pandey JP, Smith DS (2011). A Cdk5-dependent switch regulates Lis1/Ndel1/dynein-driven organelle transport in adult axons. *The Journal of Neuroscience*.

[199] Splinter D, Razafsky DS, Schlager MA (2012). BICD2, dynactin, and LIS1 cooperate in regulating dynein recruitment to cellular structures. *Molecular Biology of the Cell*.

[200] Lam C, Vergnolle MAS, Thorpe L, Woodman PG, Allan VJ (2010). Functional interplay between LIS1, NDE1 and NDEL1 in dynein-dependent organelle positioning. *Journal of Cell Science*.

[201] Liang Y, Yu W, Li Y (2004). Nudel functions in membrane traffic mainly through association with Lis1 and cytoplasmic dynein. *Journal of Cell Biology*.

[202] Mori D, Yano Y, Toyo-Oka K (2007). NDEL1 phosphorylation by Aurora-A kinase is essential for centrosomal maturation, separation, and TACC3 recruitment. *Molecular and Cellular Biology*.

[203] Vergnolle MAS, Taylor SS (2007). Cenp-F links kinetochores to Ndel1/Nde1/Lis1/dynein microtubule motor complexes. *Current Biology*.

[204] Bradshaw NJ, Soares DC, Carlyle BC (2011). Pka phosphorylation of NDE1 is DISC1/PDE4 dependent and modulates its interaction with LIS1 and NDEL1. *The Journal of Neuroscience*.

[205] Huang J, Roberts AJ, Leschziner AE, Reck-Peterson SL (2012). Lis1 acts as a, “clutch” between the ATPase and microtubule-binding domains of the dynein motor. *Cell*.

[206] Allan VJ (2011). Cytoplasmic dynein. *Biochemical Society Transactions*.

[207] McKenney RJ, Vershinin M, Kunwar A, Vallee RB, Gross SP (2010). LIS1 and NudE induce a persistent dynein force-producing state. *Cell*.

[208] Vallee RB, McKenney RJ, Ori-McKenney KM (2012). Multiple modes of cytoplasmic dynein regulation. *Nature Cell Biology*.

[209] Egan MJ, Tan K, Reck-Peterson SL (2012). Lis1 is an initiation factor for dynein-driven organelle transport. *Journal of Cell Biology*.

[210] Zheng Y, Wildonger J, Ye B (2008). Dynein is required for polarized dendritic transport and uniform microtubule orientation in axons. *Nature Cell Biology*.

[211] Taya S, Shinoda T, Tsuboi D (2007). DISC1 regulates the transport of the NUDEL/LIS1/14-3-3*ε* complex through Kinesin-1. *The Journal of Neuroscience*.

[212] Yamada M, Toba S, Yoshida Y (2008). LIS1 and NDEL1 coordinate the plus-end-directed transport of cytoplasmic dynein. *EMBO Journal*.

[213] Francis F, Koulakoff A, Boucher D (1999). Doublecortin is a developmentally regulated, microtubule-associated protein expressed in migrating and differentiating neurons. *Neuron*.

[214] Gleeson JG, Peter T L, Flanagan LA, Walsh CA (1999). Doublecortin is a microtubule-associated protein and is expressed widely by migrating neurons. *Neuron*.

[215] Horesh D, Sapir T, Francis F (1999). Doublecortin, a stabilizer of microtubules. *Human Molecular Genetics*.

[216] Fourniol FJ, Sindelar CV, Amigues B (2010). Template-free 13-protofilament microtubule-MAP assembly visualized at 8 A resolution. *Journal of Cell Biology*.

[217] Moores CA, Perderiset M, Francis F, Chelly J, Houdusse A, Milligan RA (2004). Mechanism of microtubule stabilization by doublecortin. *Molecular Cell*.

[218] Bechstedt S, Brouhard GJ (2012). Doublecortin recognizes the 13-protofilament microtubule cooperatively and tracks microtubule ends. *Developmental Cell*.

[219] Coquelle FM, Levy T, Bergmann S (2006). Common and divergent roles for members of the mouse DCX superfamily. *Cell Cycle*.

[220] Reiner O, Coquelle FM, Peter B (2006). The evolving doublecortin (DCX) superfamily. *BMC Genomics*.

[221] Sapir T, Horesh D, Caspi M (2000). Doublecortin mutations cluster in evolutionarily conserved functional domains. *Human Molecular Genetics*.

[222] Taylor KR, Holzer AK, Bazan JF, Walsh CA, Gleeson JG (2000). Patient mutations in doublecortin define a repeated tubulin-binding domain. *Journal of Biological Chemistry*.

[223] Tanaka T, Serneo FF, Higgins C, Gambello MJ, Wynshaw-Boris A, Gleeson JG (2004). Lis1 and doublecortin function with dynein to mediate coupling of the nucleus to the centrosome in neuronal migration. *Journal of Cell Biology*.

[224] Tsukada M, Prokscha A, Eichele G (2006). Neurabin II mediates doublecortin-dephosphorylation on actin filaments. *Biochemical and Biophysical Research Communications*.

[225] Tsukada M, Prokscha A, Oldekamp J, Eichele G (2003). Identification of neurabin II as a novel doublecortin interacting protein. *Mechanisms of Development*.

[226] Tsukada M, Prokscha A, Ungewickell E, Eichele G (2005). Doublecortin association with actin filaments is regulated by neurabin II. *Journal of Biological Chemistry*.

[227] Friocourt G, Chafey P, Billuart P (2001). Doublecortin interacts with *μ* subunits of clathrin adaptor complexes in the developing nervous system. *Molecular and Cellular Neuroscience*.

[228] Yap CC, Vakulenko M, Kruczek K (2012). (DCX) mediates endocytosis of neurofascin independently of microtubule binding. *The Journal of Neuroscience*.

[229] Gdalyahu A, Ghosh I, Levy T (2004). DCX, a new mediator of the JNK pathway. *EMBO Journal*.

[230] Tanaka T, Serneo FF, Tseng HC, Kulkarni AB, Tsai LH, Gleeson JG (2004). Cdk5 phosphorylation of doublecortin ser297 regulates its effect on neuronal migration. *Neuron*.

[231] Graham ME, Ruma-Haynes P, Capes-Davis AG (2004). Multisite phosphorylation of doublecortin by cyclin-dependent kinase 5. *Biochemical Journal*.

[232] Schaar BT, Kinoshita K, McConnell SK (2004). Doublecortin microtubule affinity is regulated by a balance of kinase and phosphatase activity at the leading edge of migrating neurons. *Neuron*.

[233] Bilimoria PM, De La Torre-Ubieta L, Ikeuchi Y, Becker EBE, Reiner O, Bonni A (2010). A JIP3-regulated GSK3*β*/DCX signaling pathway restricts axon branching. *The Journal of Neuroscience*.

[234] Bielas SL, Serneo FF, Chechlacz M (2007). Spinophilin facilitates dephosphorylation of doublecortin by pp1 to mediate microtubule bundling at the axonal wrist. *Cell*.

[235] Shmueli A, Gdalyahu A, Sapoznik S, Sapir T, Tsukada M, Reiner O (2006). Site-specific dephosphorylation of doublecortin (DCX) by protein phosphatase 1 (PP1). *Molecular and Cellular Neuroscience*.

[236] Tint I, Jean D, Baas PW, Black MM (2009). Doublecortin associates with microtubules preferentially in regions of the axon displaying actin-rich protrusive structures. *The Journal of Neuroscience*.

[237] Cohen D, Segal M, Reiner O (2007). Doublecortin supports the development of dendritic arbors in primary hippocampal neurons. *Developmental Neuroscience*.

[238] Moores CA, Perderiset M, Kappeler C (2006). Distinct roles of doublecortin modulating the microtubule cytoskeleton. *EMBO Journal*.

[239] Liu JS, Schubert CR, Fu X (2012). Molecular basis for specific regulation of neuronal kinesin-3 motors by doublecortin family proteins. *Molecular Cell*.

[240] Pramparo T, Libiger O, Jain S (2011). Global developmental gene expression and pathway analysis of normal brain development and mouse models of human neuronal migration defects. *PLoS Genetics*.

[241] Pramparo T, Youn YH, Yingling J, Hirotsune S, Wynshaw-Boris A (2010). Novel embryonic neuronal migration and proliferation defects in Dcx mutant mice are exacerbated by Lis1 reduction. *The Journal of Neuroscience*.

[242] Gambello MJ, Darling DL, Yingling J, Tanaka T, Gleeson JG, Wynshaw-Boris A (2003). Multiple dose-dependent effects of Lis1 on cerebral cortical development. *The Journal of Neuroscience*.

[243] Youn YH, Pramparo T, Hirotsune S, Wynshaw-Boris A (2009). Distinct dose-dependent cortical neuronal migration and neurite extension defects in Lis1 and Ndel1 mutant mice. *The Journal of Neuroscience*.

[244] Silver DL, Watkins-Chow DE, Schreck KC (2010). The exon junction complex component Magoh controls brain size by regulating neural stem cell division. *Nature Neuroscience*.

[245] Hebbar S, Mesngon MT, Guillotte AM, Desai B, Ayala R, Smith DS (2008). Lis1 and Ndel1 influence the timing of nuclear envelope breakdown in neural stem cells. *Journal of Cell Biology*.

[246] Hippenmeyer S, Youn YH, Moon HM (2010). Genetic mosaic dissection of Lis1 and Ndel1 in neuronal migration. *Neuron*.

[247] Yingling J, Youn YH, Darling D (2008). Neuroepithelial stem cell proliferation requires lis1 for precise spindle orientation and symmetric division. *Cell*.

[248] Pawlisz AS, Mutch C, Wynshaw-Boris A, Chenn A, Walsh CA, Feng Y (2008). Lis1-Nde1-dependent neuronal fate control determines cerebral cortical size and lamination. *Human Molecular Genetics*.

[249] Cahana A, Escamez T, Nowakowski RS (2001). Targeted mutagenesis of Lis1 disrupts cortical development and LIS1 homodimerization. *Proceedings of the National Academy of Sciences of the United States of America*.

[250] Hirotsune S, Fleck MW, Gambello MJ (1998). Graded reduction of Pafah1b1 (Lis1) activity results in neuronal migration defects and early embryonic lethality. *Nature Genetics*.

[251] Shu T, Ayala R, Nguyen MD, Xie Z, Gleeson JG, Tsai LH (2004). Ndel1 operates in a common pathway with LIS1 and cytoplasmic dynein to regulate cortical neuronal positioning. *Neuron*.

[252] Umeshima H, Hirano T, Kengaku M (2007). Microtubule-based nuclear movement occurs independently of centrosome positioning in migrating neurons. *Proceedings of the National Academy of Sciences of the United States of America*.

[253] McManus MF, Nasrallah IM, Pancoast MM, Wynshaw-Boris A, Golden JA (2004). Lis1 is necessary for normal non-radial migration of inhibitory interneurons. *American Journal of Pathology*.

[254] Moore KD, Chen R, Cilluffo M, Golden JA, Phelps PE (2012). Lis1 reduction causes tangential migratory errors in mouse spinal cord. *Journal of Comparative Neurology*.

[255] Corbo JC, Deuel TA, Long JM (2002). Doublecortin is required in mice for lamination of the hippocampus but not the neocortex. *The Journal of Neuroscience*.

[256] Reiner O, Gorelik A, Greenman R (2012). Use of RNA interference by in utero electroporation to study cortical development: the example of the doublecortin superfamily. *Genes*.

[257] Kappeler C, Saillour Y, Baudoin JP (2006). Branching and nucleokinesis defects in migrating interneurons derived from doublecortin knockout mice. *Human Molecular Genetics*.

[258] Friocourt G, Liu JS, Antypa M, Rakić S, Walsh CA, Parnavelas JG (2007). Both doublecortin and doublecortin-like kinase play a role in cortical interneuron migration. *The Journal of Neuroscience*.

[259] Bai J, Ramos RL, Paramasivam M, Siddiqi F, Ackman JB, LoTurco JJ (2008). The role of DCX and LIS1 in migration through the lateral cortical stream of developing forebrain. *Developmental Neuroscience*.

[260] Ocbina PJ, Dizon MLV, Shin L, Szele FG (2006). Doublecortin is necessary for the migration of adult subventricular zone cells from neurospheres. *Molecular and Cellular Neuroscience*.

[261] Greenwood JSF, Wang Y, Estrada RC, Ackerman L, Ohara PT, Baraban SC (2009). Seizures, enhanced excitation, and increased vesicle number in Lis1 mutant mice. *Annals of Neurology*.

[262] Jones DL, Baraban SC (2009). Inhibitory inputs to hippocampal interneurons are reorganized in Lis1 mutant mice. *Journal of Neurophysiology*.

[263] Hunt RF, Dinday MT, Hindle-Katel W, Baraban SC (2012). LIS1 deficiency promotes dysfunctional synaptic integration of granule cells generated in the developing and adult dentate gyrus. *The Journal of Neuroscience*.

[264] Valdés-Sánchez L, Escámez T, Echevarria D (2007). Postnatal alterations of the inhibitory synaptic responses recorded from cortical pyramidal neurons in the Lis1/sLis1 mutant mouse. *Molecular and Cellular Neuroscience*.

[265] Kawabata I, Kashiwagi Y, Obashi K (2012). LIS1-dependent retrograde translocation of excitatory synapses in developing interneuron dendrites. *Nature Communications*.

[266] Nosten-Bertrand M, Kappeler C, Dinocourt C (2008). Epilepsy in Dcx knockout mice associated with discrete lamination defects and enhanced excitability in the hippocampus. *PLoS ONE*.

[267] Kerjan G, Koizumi H, Han EB (2009). Mice lacking doublecortin and doublecortin-like kinase 2 display altered hippocampal neuronal maturation and spontaneous seizures. *Proceedings of the National Academy of Sciences of the United States of America*.

[268] Bazelot M, Simonnet J, Dinocourt C (2012). Cellular anatomy, physiology and epileptiform activity in the CA3 region of Dcx knockout mice: a neuronal lamination defect and its consequences. *European Journal of Neuroscience*.

[269] Ackman JB, Aniksztejn L, Crépel V (2009). Abnormal network activity in a targeted genetic model of human double cortex. *The Journal of Neuroscience*.

[270] Lapray D, Popova IY, Kindler J (2010). Spontaneous epileptic manifestations in a DCX knockdown model of human double cortex. *Cerebral Cortex*.

[271] Yamada M, Yoshida Y, Mori D (2009). Inhibition of calpain increases LIS1 expression and partially rescues in vivo phenotypes in a mouse model of lissencephaly. *Nature Medicine*.

[272] Yamada M, Hirotsune S, Wynshaw-Boris A (2010). A novel strategy for therapeutic intervention for the genetic disease: preventing proteolytic cleavage using small chemical compound. *International Journal of Biochemistry and Cell Biology*.

[273] Manent JB, LoTurco J, Noebels JL, Avoli M, Rogawski MA, Olsen RW (2012). Reversing disorders of neuronal migration and differentiation in animal models. *Jasper's Basic Mechanisms of the Epilepsies*.

[274] Sebe JY, Bershteyn M, Hirotsune S, Wynshaw-Boris A, Baraban SC (2013). ALLN rescues an in vitro excitatory synaptic transmission deficit in Lis1 mutant mice. *Journal of Neurophysiology*.

[275] Manent JB, Wang Y, Chang Y, Paramasivam M, LoTurco JJ (2009). Dcx reexpression reduces subcortical band heterotopia and seizure threshold in an animal model of neuronal migration disorder. *Nature Medicine*.

